# Efficacy of photodynamic therapy and periodontal treatment in patients with gingivitis and fixed orthodontic appliances: Protocol of randomized, controlled, double-blind study

**DOI:** 10.1097/MD.0000000000019429

**Published:** 2020-04-03

**Authors:** Ellen Perim Rosa, Felipe Murakami-Malaquias-Silva, Tânia Oppido Schalch, Daniela Bezerra Teixeira, Ricardo Fidos Horliana, Andre Tortamano, Isabel Peixoto Tortamano, Inês Aparecida Buscariolo, Priscila Larcher Longo, Renata Matalon Negreiros, Sandra Kalil Bussadori, Lara Jansiski Motta, Anna Carolina Ratto Tempestini Horliana

**Affiliations:** a Postgraduate Program in Biophotonics Applied to Health Sciences, Universidade Nove de Julho, UNINOVE, São Paulo, Brazil; b Academic specialization student in Temporomandibular Disorder and Orofacial pain; c Department of Orthodontics, School of Dentistry, University of São Paulo, São Paulo, SP, Brazil; d Department of Stomatology, School of Dentistry, University of São Paulo, São Paulo, SP, Brazil; e Postgraduate Program of Aging Science -São Judas Tadeu University - São Paulo, SP, Brazil.

**Keywords:** gingivitis, orthodontic appliance, photodynamic therapy

## Abstract

It is known that the presence of orthodontic brackets predisposes for a change in the biofilm, facilitating the development of gingivits. The sites are difficult to access with a toothbrush and periodontal curette, worsening inflammation, in addition, a gingival hyperplasia is associated with poor hygiene. The objective of this study is to evaluate the impact of photodinamyc therapy (PDT) as an adjuvant treatment, considering clinical immunoregulatory and microbiological parameters. This randomized, controlled, double-blind clinical study will include 34 patients, both genders, having used fixed appliance for more than 12 months, with gingivitis. Participants will be divided into two groups: G1 (n = 17)- Scaling and Root Planing + PDT placebo and G2 (n = 17)- Scaling and Root Planing + PDT. In G2 the following dosimetric parameters will be used: methylene blue 0.005%, λ= 660 nanometers (nm), 9 Joules (J) per site, irradiance= 3.5Watts (W)/ centimeters (cm), radiant exposure= 318J/cm^2^. All participants will receive oral hygiene guidance prior the curetes scaling. The clinical periodontal data to be analyzed are plaque index, gingival index and probing depth. Crevicular fluid, from 4 pre-determined sites and saliva, will be collected and analysed for IL-6, IL-1β, IL-8, TNF-α and IL-10 cytokines using ELISA (Enzyme immunoabsorption assay) method. Total *Bacteria* count will also be performed, by qPCR and *Universal16SrRNA* gene. All analysis will be realized using in the baseline (T_0_), 7 (T_1_) and 21 (T_2_) days after treatment. Oral health-related quality of life will be assessed using the OHIP-14 questionnaire at times T_0_ and T_2_. If sample distribution is normal, the Student T-test will be applied if it is not normal, the Mann-Whitney test will be used. The data will be presented in terms of ± PD and The significance level will be set at p < 0.05. Our results may improve quality of life and add data to establish a therapeutic alternative for gingivitis during the orthodontic treatment. Registration: clinicaltrials.gov NCT04037709. https://clinicaltrials.gov/ct2/show/NCT04037709 - Registered in July 2019.

## 1 Introduction

It is known that the presence of fixed orthodontic devices - brackets, bands and orthodontic elastics, facilitate the biofilm accumulation due to the difficulty in properly brushing, besides favoring the change in the quantity and quality of biofilm.^[1,2,3]^ Retentive areas such as brackets are favorable for microbiological aggregation and thus for the inflammatory process in local tissues.^[4,5]^

Biofilm accumulation occurs in brackets of various materials, such as stainless steel, gold, ceramic and plastic,^[6]^ whether conventional or self-bonding.^[7]^ For Jurela A, et al, 2013^[8]^ there was no difference comparing microorganisms in saliva of users of brackets composed of different materials, for Pandis N, et al, 2010^[7]^ there was also no difference in microorganisms in saliva with two different types of devices: conventional and self-ligating. Regarding gum health, ceramic brackets appear to have better results, especially for adolescent patients, which correspond to most users of multi-brace orthodontics.^[9]^ Microbiological adhesion to brackets is a predisposing factor to development of gingivitis and it increases the retentive surface. Inflammatory changes are frequently observed in the presence of non-removable orthodontic devices in patients with dentomaxillary anomalies. These parameters can be clinically and chemically evaluated, Bracket placement was followed by deterioration in oral hygiene, increased total protein levels, hydrogen sulfide and nitrogen metabolites in oral fluid.^[3]^ Microscopic analysis demonstrates the direct effect of bracket bonding on the middle third of the buccal surface of the tooth crown, where the bonding area even after bracket removal has numerous areas of disordered grooves and fissures, which may facilitate the retention of microorganisms. These areas are very different from the smooth surface of the lingual faces, for example, which do not receive any type of bonding material. Therefore, caution in bonding is required to avoid overeating as well as clinical alternatives in fixed fixture removal and proper polishing procedures.^[10]^

Oral hygiene is one of the most important questions for patients in orthodontic care, since the presence of fixed devices (brackets and bands) and removable devices (wires and elastics) decreases the efficiency of tooth brushing, especially in the interproximal areas, causing high measurements in the plaque index (PI) and gingival index (GI).^[11]^

Biofilm accumulation is better controlled in those patients who have the habit of brushing their teeth two or more times a day, using interdental brushes, flossing and having frequent consultations in a short time. The rate of PI and gingival bleeding in patients who control oral hygiene during orthodontic treatment is significantly lower. To maintain gum health and reduce the amount of biofilm adhered to orthodontic appliances, patients should be constantly informed about the merits of oral hygiene brushing.^[11,12]^

Periodontal health is defined by the absence of clinically detectable inflammation. Gingival homeostasis can be recovered in individuals who have already had gingivitis and the fact that there is no bone tissue involvement facilitates the return to the pre-pathogenic condition.^[13]^ Clinically, the healthy periodontium is characterized by the absence of bleeding, erythema and edema, physiological with bone levels range from 1.0 to 3.0 mm apical to the enamel-cement junction (ECJ) line. In the transition from periodontal health to gingivitis, ≥10% of bleeding sites and clinical probing depth greater than 3.0 mm can be observed. However, this probing depth greater are gingival hyperplasia, a condition where the increase in gingival tissue above the ECJ line forms a deeper pocket, unlike periodontitis, where the increase in clinical probing depth occurs due to bone loss below the ECJ line. In cases of gingival hyperplasia there is also a possibility of recovering the health and appearance of the gum, because if the regression is not spontaneous, surgical removal may be used.^[13]^ For clinical diagnosis, the gingival bleeding rate is a relevant parameter, where a tube exerts slight pressure on the gingival margins and bleeding can occur immediately or within seconds.^[14]^

Gingivitis can be classified as biofilm-induced or non-biofilm-induced. Those biofilm-induced may be: a) associated with dental biofilm only, b) mediated by local and systemic risk factors or c) drug-influenced gingival enlargement.^[15]^ Those non-biofilm-induced is caused by systemic factors, regardless of the presence of biofilm, such as genetic, hormonal, immune factors, among others.^[16]^ From a microbiological point of view, we have the contemporary model of host - microorganism interaction in the pathogenesis of periodontitis, where the host response drives an incipient dysbiosis (gingivitis). If the biofilm is not interrupted/removed, blunt dysbiosis results and perpetuates chronic and destructive inflammation. In the presence of incipient dysbiosis, the host may produce, polymorphonuclear neutrophils, T and B defense cells, antibodies and with the progression of inflammation due to the high amount of biofilm there is also production of antigens, perpetuation of microorganisms with high virulence and lipopolysaccharides.^[13]^ Thus, biofilm is considered a determining factor for gingival inflammation and its severity depends on local and systemic factors.^[15]^

Some periodontopathogens are well known to be present in the destruction of periodontal tissues, such as *Aggregatibacter actinomycetemcomitans*, *Porphyromonas gingivalis* and *Fusobacteria nucleatum*. The *Prevotella intermedia* was also correlated as an anaerobic bacterium present in biofilm samples from patients using fixed orthodontic appliance.^[17]^ However, emerging evidence suggests a clustering of bacteria rather than individual species. Actually, the diagnosis through biological markers have been considered more robust in discriminating bacterial functions such as proteolysis, flagella and bacterial motility. There is the characterization of the disease and not of a species, so the presence of a spare species is not considered a pathognomonic sign.^[14]^

Currently biomarkers have also been used in the diagnosis of gingivitis. Inflammatory cytokines from crevicular fluid and saliva have been evaluated by ELISA^[18]^ real time polymerase chain reaction (PCR) method^[19]^ For the treatment of gingivitis, the gold standard is usually biofilm scraping, usually performed with gracey curettes and/or ultrasonic equipment.^[20]^

The major drawback in treating gingivitis with conventional methods in the patient with brackets and bands is that there may be gingival hyperplasia covering the upper base toward the cervical and upper fins, ideally to perform instrumentation around the entire bracket until the amelocemental line, considering the potential for biofilm accumulation. Comparing the angles of piezoelectric ultrasound in biofilm removal brackets was performed around bracket only at the occlusal base and it was shown that a prolonged ultrasonic instrumentation should not be performed at the bracket base, due to the possibility of detachment^[20]^

Mechanical removal of gold standard biofilm in the treatment of periodontal diseases is impaired in hard to reach areas because of the difficulty in entering small areas. The Photodynamic Therapy (PDT) has been shown to be promising as adjunctive therapy to periodontal treatments with regard to its antimicrobial action, the technique consists of the use of low wavelength lasers associated with a photosensitizing agent.^[22]^ PDT has no bacterial selectivity and side effects such as bactericidal/ bacteriostatic, preserves the oral microbiota and has no anti-inflammatory toxicity.^[23]^ There is a triad for PDT to happen: photosensitizing agent, light and oxygen.^[24]^ Photosensitizing agent has the property of selective accumulation in both normal and infected tissues, it is able to penetrate damaged and dead tissues without developing selectivity.^[25]^ The photosensitizing agent is composed of cationic molecules that have low molecular weight, when inserted in a pouch has the ability to quickly bind to local bacteria.^[26]^ Methylene blue (MB) is the most widely used^[27,28]^ in Brazil, being the only one that has ANVISA approval. To provide the antimicrobial effect, the dye is associated with a light (low power laser), establishing a photochemical effect, which occurs due to the presence of molecules sensitive to certain wavelengths. There is a photoregulation, changing the functional and metabolic activity of the irradiated cell.^[29]^ MB has wavelength absorption from 600 to 660 nm, being the appropriate spectrum for the therapeutic window.^[30]^ The therapeutic window of PDT is effective in eradicating colonies or biofilm and may intensify the results of conventional mechanical treatment.^[25,31]^ The oxygen molecules of the Photosensitizing when they excited by light irradiation, originate reactive oxygen species, such as singlet oxygen, which is capable of causing selective bacterial death.^[26]^ Oxygen is the third element of the fundamental triad in PDT. The joint use of periodontal treatment and PDT has been considered in reducing the extent of inflammatory gum signs. To evaluate laser effectiveness in this context, it is important to consider a placebo group, making the study blind. Furthermore, the experimental hypothesis is aimed at assessing whether PDT associated with periodontal treatment is effective in the treatment of gingivitis (gingival hyperplasia) caused by poor hygiene associated with orthodontic braces in healthy young patients.

## 2 Materials and methods

This is a single-center randomized, double-blind, prospective, controlled trial with 21 days of follow-up. A revised version of the manuscript has been submitted, and SPIRIT-Checklist and SPIRIT-Figure. The project was submitted to the Research Ethics Committee (REC) of the Universidade Nove de Julho – UNINOVE and approved (#3505689). After verbal and written explanation of the study, participants who agree to participate in the study will sign an Informed Consent Form (ICF) and a Terms of Assent (TA) for children between 10–18 years of age, the person responsible for applying the therm will be the researcher (EPR). The study will be conducted in accordance with the *Declaration of Helsinki* (revised in Fortaleza, 2013). The Project was registered at www.clinicaltrial.gov (NCT 04037709). The patients will come from three centers located in Brazil: Dellê Dentistry – 23 Marília de Dirceu Street, Jardim Aeroporto - São Paulo; Odontocorpus Dentistry and Health – 16 Dr. Antonio Ruggiero Junior Street, Pirituba- São Paulo; and Fernanda Dias and Team Clinic– 761 Mariana Ubaldina do Espírito Santo Avenue, Bom Clima – Guarulhos, from July 2019 to December 2021. There is no conflict of interest regarding the clinics where the research will be conducted. Project receive grant from Brazilian National Coordination for the Improvement of Higher Education Personnel CAPES # 1690040 (CAPES Portuguese: Coordenação de Aperfeiçoamento de Pessoal de Nível Superior (CAPES). Any intercurrence will be notified to the REC and reported in the publication. If necessary, the study will be ended before the stated date. In addition, if evaluations of the dosimetric parameters for the laser, as described in the initial protocol, show that the results are not adequate, the protocol may be adjusted. The sample will be composed of patients who have used a fixed appliance for at least 12 months and with gingivitis present, detailed description in the sample. The experimental design will consist of 2 groups (described in detail in Experimental Design). Samples will be collected for microbiological evaluation and measurement of inflammatory cytokines in the crevicular fluid and saliva. Periodontal clinical data will be collected for diagnosis of gingivitis and for comparison of these parameters after 7 and 21 days to check for improvements. The Oral Health Impact Profile Questionnaire (OHIP-14) questionnaire will also be applied to measure the quality of life related to oral health.

All data will be published without any type of restriction and will be made available. If participants wish to receive the research data they can give their e-mail and a copy of the article will be sent to them as soon as it is published. Furthermore, all data collected will be kept confidential.

### 2.1 Calculation of sample size

To achieve an effect size = 0.40 between two groups (conventional treatment and PDT), for the difference in the amount of bacteria present in the crevicular fluid of participantstreated with scaling and those treated with PDT, assuming a type 1 error of 0.2^[1]^ and 95% confidence level, the total sample size will be 34 participantsdistributed into 2 groups. The sample calculation was performed in Excel, based on the calculation formula described by Kadam and Bhalerao. Considering a 20% drop-out, 3 extra participantswill be inserted into each group, if there is said drop-out this will provide a final sample of participants per group.^[32]^

To ensure participants’ adherence to the research, the steps will preferably coincide with the participants's return visits to their routine orthodontic appliance maintenance. Participants uptake will occur during routine consultations with the orthodontist.

### 2.2 Description of the sample

The sample will be composed of healthy participants being treated with a fixed orthodontic appliance for at least 12 months and who present with gingivitis (GI greater than 10%, probing depth ≤ 3 mm from the distance from the ECJ line to the bottom of the periodontal pocket, pseudopockets will be included in the study). We will also consider the clinical signs of gingival inflammation: edema (loss of sharp gingival margin, blunting of the papillae and hyperplasia), erythema and probing discomfort. Relevant patient complaints will be considered to begin the anamnesis: gingival bleeding (metallic taste / altered taste), pain, halitosis and difficulties when eating.^[13]^

### 2.3 Inclusion / exclusion criteria

To be included: healthy participants (negative medical history), of both genders, aged 10 to 30 years, with gingivitis induced by dental biofilm, predisposed by the use of a fixed orthodontic appliance, according to the classification of 2018.^[33]^

To be excluded: participants with maxillary and mandibular deformities, periodontitis, oral lesions and who have used antibiotics for less than 3 months, those who have used non-steroidal anti-inflammatory drugs and continuous corticosteroid therapy^[1]^ for less than 3 months, as well as those who have been using a mouthwash in the past 3 months. Participants who have modifying factors for periodontal disease, such as diabetics, immunosuppressive smokers (cyclosporine), anticonvulsants (phenytoin), calcium channel blockers (nifedipine) and pregnant and lactating women as well as those who are HIV positive, or have Hepatitis B or C. Patients who require prophylactic antibiotic therapy for periodontal treatment who have had periodontal treatment in the last 6 months. Patients who have non-biofilm-induced gum disease.^[33]^ Patients who for some reason during the study have to initiate the use of the medicines and mouthwashes mentioned above or acquire a disease that is a modifying factor for periodontal disease or become pregnant. Patients who do not wish to remain part of the study may drop out whenever they wish.

### 2.4 Researcher calibration

For calibration purposes one examiner will evaluate 5 patients with gingivitis, who will not be part of the study. Two complete periodontal exams (except third molars) per patient (with a 30-minute interval between them) will be performed at six sites per tooth, for clinical probing depth (PD), clinical level of insertion, PI and GI. The intra-examiner calibration will be repeated every 4 months. The intraclass correlation coefficient (ICC) will be calculated in order to evaluate the intra-examiner concordance ≥ 0.75 in relation to clinical periodontal parameters. The evaluation will be performed with periodontal probe marked in millimeters and this researcher will not be involved in the treatment of patients. This same examiner will be trained for biofilm and saliva collections for cytokine and microbiological analysis to maximize the reproducibility of the evaluations.

### 2.5 Randomization

To randomly distribute the participants into the experimental groups, a 40-lot draw will be made using the Microsoft Excel program, version 2017. The distribution of groups will be identical (1: 1) for the two groups. The distribution will be done in a blocking fashion (8 groups of 5 patients). Opaque envelopes will be identified with sequential numbers (1 to 40) and there will be information about the corresponding experimental group in the order obtained in the draw. The envelopes will be sealed and will remain sealed in numerical order until the start of the periodontal treatments. The drawing and preparation of the envelopes will be performed by a person not involved in the study. Immediately prior to periodontal treatment the investigator responsible for treatment will open 1 envelope (without changing the numerical sequence) and perform the indicated procedure. Three additional patients will be included in each group in view of the dropout predicted in any clinical study (considering a dropout of 20%).

### 2.6 Study blinding

Only the researcher responsible for conducting the treatments (who will open the randomized envelopes) will know which treatment is assigned to each patient. The identification of each group will only be revealed by this researcher after statistical analysis of the data for all those involved in the study. Therefore, the researcher responsible for data collection, the microbiologist and the statistician will be blind regarding the treatments assigned to the groups. The patient will also be blind to the type of treatment performed, since the periodontal treatment will be identical in both groups and treatment with PDT will be simulated in the control group.

### 2.7 Pretreatment evaluations

Participants will sign the ICF, TA (when necessary), the anamnesis, periodontal clinical examination and the OHIP-14 questionnaire will be done. These data will be collected by 1 calibrated researcher. Collection of saliva and biofilm from all patients will be performed to evaluate the inflammatory cytokines (according to item ‘Salivary and biofilm cytokines profile’) and for microbiological evaluation (according to the item “Microbiological analysis”). Following which, OHG will be given to all patients, and then the treatment in accordance with the randomization.

### 2.8 Anamnesis

Anamnesis will be performed in both groups. In addition to general patient health questions, demographic data (age, gender, marital status, occupation, educational level, living conditions, salary) and medical history data (primary complaint, current illness status, medical history, dental history, and medications) will be collected.

### 2.9 Methodology used for the clinical evaluation of gingivitis

It will be performed by an evaluator calibrated with periodontal probe in millimeters (Periodontal probe from the University of North Carolina UNC-15 - Hu-Friedy^TM^). The periodontal probe will be inserted at all predetermined sites at the gingival margin of all teeth, 30 seconds will be waited and, at the smallest sign of bleeding, the presence or absence of bleeding will be noted, in a dichotomous manner, for that site.^[34]^ Gingival bleeding will be evaluated at 6 sites (mesiobuccal, buccal, distobuccal, mesiolingual, lingual, distolingual).

- Gingival index:

Presence (1) of bleeding with probing at gingival margin,^[34]^ or

Absence (0) of bleeding with probing at gingival margin.^[34]^

The gingival Index will be presented in a percentage (%). The result will be based on the ratio of total sites to sites affected (with bleeding)^[34]^

Results will be considered indicative of gingivitis with ≥ 10% bleeding.^[13]^

### 2.10 Experimental design

Immediately prior to periodontal treatment, the investigator will remove and open 1 envelope (without changing the numerical sequence of the other envelopes) and perform the indicated procedure. In this manner, the 34 patients will be allocated into the experimental or control groups, as follows:

**G1**- Control group (n = 17) - patients will receive scaling and oral hygiene guidance + application of placebo PDT.

**G2**- Experimental group (n = 17) - patients will be treated with scaling and oral hygiene guidance + PDT application. Photodynamic therapy will be performed at 8 sites with gingival hyperplasia (pseudopocket).

### 2.11 Oral hygiene guidance (OHG)

Immediately prior to treatment all participants will receive OHG: BASS brushing technique, specifications for toothbrush (soft bristles, only two rows for hygiene below and above brackets, narrow, rectangular head), interdental brush (0.05 mm) and dental floss (with wax attached to the thread).

To ensure that participants are adhering to the guidelines, they will be asked to bring their brushes and floss to check if the brushing technique is being performed correctly.

At all meetings the participant will be questioned about the use of mouthwashes as it is prohibited during the study. If use is reported, the participant will be excluded from the study.

### 2.12 Treatments

#### 2.12.1 Periodontal treatment

Participants in both (G1 and G2) groups will receive periodontal treatment (scaling and root planing - SRP) with universal curettes (Hu-Friedy) and ultrasound (Ultrassom Dabi Atlante - Profi Neo US, Ribeirão Preto, Brazil) depending on their needs. The SRP will be done in one session. Periodontal treatment will be performed by only one experienced researcher, who will not do the periodontal exams. Periodontal reassessment will be performed after 7 and 21 days. All patients in the control group will receive SRP, but will receive only a simulation of photodynamic therapy. To remove supragingival dental biofilm the Gracey Curette #3-4 will be used for the free surfaces of the anterior teeth and #7-8 on the free surfaces of the posterior teeth.

#### 2.12.2 Photodynamic therapy

Group 2 participants will receive PDT; the technique associates a photosensitive agent with a source of light and oxygen, in order to reduce the microbial load in places that are not accessible by curette.

After scaling, the procedures will be performed as described:

- Application of methylene blue (0.005% - Chimiolux 5, DMC - purified water and methylene blue) with a carpule syringe and needle (with stop and without bevel) into the pseudopockets;
  - Wait 1 minute (pre-irradiation time)^[35]^
- Irradiation with a red laser diode (λ = 660 nm) with an output power of 100mW (Therapy EC, DMC, São Paulo, SP, Brazil). The laser head will be positioned in direct contact with the pseudo periodontal pocket.^[36]^
  - Apply at 8 sites where there is hyperplasia.
  - Wash in abundance with saline solution until the photosensitizer is completely removed.
  - During laser application both patient and operator will wear safety goggles.

Participants in G1 (Control- PDT placebo) will receive an agent with the same vehicle as that of the methylene blue to mimic irrigation with the photosensitizer; the laser will be switched off at the time of application.

PDT placebo procedures will be performed after scaling. The main difference between G1 and G2 is that in G1 the lase will be turned off but the same device will be positioned in the same way and with the same time of G2. Also, the beep sound will be recorded and turned on during application.

**Table 1:**
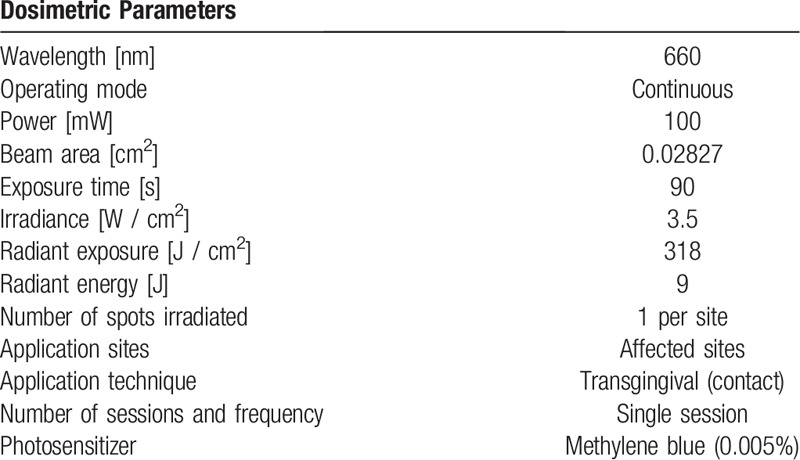
Parameters used for PDT.

### 2.13 Study variables

The primary study variable will be

- GI as determined using a periodontal probe at baseline (T_0_), 7 (T_1_) and 21 (T_2_) days after treatment.

The secondary variables of the study will be:

- Clinical PD at baseline (T_0_), 7 (T_1_) and 21 (T_2_) days after treatment;
- Visible PI at baseline (T_0_), 7 (T_1_) and 21 (T_2_) days after treatment;
- GI at baseline (T_0_), 7 (T_1_) and 21 (T_2_) days after treatment.
- Oral health-related quality of life (OHRQoL) will be assessed using the OHIP-14 questionnaire at baseline (T_0_) and 21 (T_2_) days after treatment;
- Evaluation of cytokines IL-6, IL-1β, IL-8, TNF-α and IL-10, by ELISA method, in non-stimulated saliva and crevicular fluid at baseline (T_0_), 7 (T_1_) and 21 (T_2_) days after treatment;
- Microbiological analysis by means of the *Universal bacteria* count (16S rRNA gene) present in crevicular fluid (sites with hyperplasia), and non-stimulated saliva at baseline (T_0_), 7 (T_1_) and 21 (T_2_) days after treatment.

### 2.14 Variables

#### 2.14.1 Clinical probing depth

It will be performed by a calibrated evaluator using a periodontal probe marked in millimeters (North Carolina University periodontal probe, UNC-15 (Hu-Friedy^TM^): all teeth (except third molars) will be evaluated at 6 sites (mesiobuccal, buccal, distobuccal, mesiolingual, lingual, distolingual) using the aforementioned parameters.

The North Carolina University periodontal probe will be insert in the gingival sulcus. Probing depths to be evaluated in millimeters (mm) from the base of the periodontal pocket to the free gingival margin.

#### 2.14.2 Visible plaque index

It will be visualy evaluated before of probing depht, using the following score of visible supragingival plaque: Presence = 1; Absence = 0

#### 2.14.3 Gingival index

It will be evaluated by recording the presence or absence of bleending up to 30 seconds after the determination of probing depht. The number of positive sites bleeding will be record and than expressed as percentage of the number of sites examined. Using the following score of bleeding when probing gingival margin: Presence = 1; Absence = 0

#### 2.14.4 Analysis of oral health-related quality of life - OHIP-14

The OHIP-14 questionnaire is a simplified form of the original OHIP-49 questionnaire and will be used to assess the impact of oral health on the quality of life of the research participants. The OHIP-14 is used to measure perceived needs; evaluating the impact of oral changes on Oral health-related quality of life. The patient responds to 14 questions by assigning the values:

- 0 (never),
- 1 (almost never),
- (sometimes),
- (most of the time),
- (always) to his or her answers.

In this study it will be evaluated the OHIP-14 using the additive method and domain evaluation. In the additive method, when the value is greater than 1, one can consider the presence of a negative impact on the life of the individual. OHIP-14 will be assessed at baseline and after 3 weeks for all subjects.

#### 2.14.5 Microbiological analysis

##### 2.14.5.1 Biofilm collection

Biofilm samples from regions with gingivitis and gingival hyperplasia (pseudo pocket) will be collected from the supragingival portion to avoid contamination of the sample. The site is isolated with cotton rolls. The paper cone is inserted until resistance is felt. The cone will remain in this position for 30 seconds. If there is blood contamination a new cone will be used after 90 seconds. Five cones must be collected, one for each cytokine. The cones will be placed in a 1.5 ml microcentrifuge tube (Eppendorf) and stored at -80°C. During collection they will be properly identified and stored on ice inside a Styrofoam container. The collected samples will be stored at -80°C until further analysis is performed.

##### 2.14.5.2 Saliva collection

Samples of unstimulated saliva (5 ml) will be collected in 50 ml tubes (Falcon tube). In the laboratory 500 μl of saliva will be added to 500 μl TE, and stored at −80° C.

##### 2.14.5.3 DNA extraction

DNA extraction will be performed using Meta-G-Nome DNA Isolation - MGN0910 (Epicenter Technologies Corp. Chicago, IL, USA) according to the manufacturer's instructions. For saliva and biofilm samples, 100 μl of sample, previously vortexed at 12857 g (10,000 RPM), 4° C, for 10 minutes, will be transferred to new micro centrifuge tubes. The supernatant will be discarded, leaving 25 μl, along with the precipitate. Samples will be vortexed for resuspension of the pellet. For each sample a mixture of 2 μl of proteinase K and 300 μl of TCL (tissue and cells lysis) will be added; the samples will then be placed in a water bath for 15 minutes and vortexed every 5 minutes. After the water bath, the samples will be placed on ice for 5 minutes. 150 μl of protein precipitation reagent will be added and the samples will be vortexed for 5 seconds. They will then be centrifuged for 10 minutes, at 4°C, at 10,000 RPM. The supernatant will be discarded, leaving only the pellet. 500 μl of isopropanol will be added and the samples will be poured 40 times. Then the samples will be centrifuged again for 10 minutes, at 4°C, at 4,000 RPM, and then decanted to remove the isopropanol and leave only the pellet. 200 μl of 70% ethanol will be added and the samples will be centrifuged for 2 minutes, at 4°C and 4,000 RPM. The ethanol will be removed and the last step will be repeated. The tubes will be left open, inverted until dry, for about 30 minutes. After drying, the resulting pellet will be diluted in TE. After DNA extraction, the samples will be stored in a freezer at −20° C and quantified through the use of a spectrophotometer (NanoDrop ND1000 - Thermo Fisher Scientific Inc.).

##### 2.14.5.4 Real-time PCR

Quantification of the bacteria will be performed using real-time qPCR using the thermal cycler StepOnePlus Real-Time PCR System (Applied Biosystem, Foster City, CA, USA). The products will be detected via fluorescence using the Quantimix Easy SYG Kit (Biotools, Madrid, Spain), following the protocol recommended by the manufacturer. The following primers will be used for the reaction (Table 2):

**Table 2:**
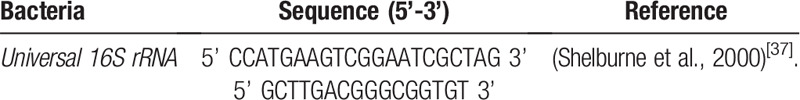
Sequences and references for primers used.

To determine the number of plasmid copies/ μl, necessary to assemble the qPCR curve, it will be estimated that 1 μg of 100 bp of DNA would be equivalent to 9x10^11^ plasmids. These will then be serially diluted, and the curve will comprise 10 to 10^8^ plasmids, which will then be compared to the DNA of the analyzed samples. The number of *Universal Bacteria* will be calculated assuming 1 copy of the 16S rRNA gene/ chromosome.^[37]^ As a negative control sterile Milli-Q water will be added instead of DNA to the template.

Quantitative analysis will be performed by comparing the results obtained with the samples to the data obtained with the standard curve. For the quantification of *Universal Bacteria* 16S rRNA in saliva and biofilm samples, species-specific primers (5 ’CCATGAAGTCGGAATCGCTAG 3” and 5’ GCTTGACGGGCGGTGT 3’) will be used.^[37]^ The reaction will be realized with a total volume of 10 μL containing 5 μL of SYBR Green, 2 μL of sample and 200 μM of each primer for 16S rRNA. Cyclization will be performed: 95°C / 10 min holding, 40 cycles at 95°C / 15 s, 60°C / 1 min, melting curve 95° C / 15 s, 60°C / 1 min (+0.6°C collection) and 95^o^C / 15 s. Only those reactions in which the efficiency was 100% (+ or - 10) R^2^ near to 1 will be considered.

##### 2.14.5.5 Analysis of salivary cytokine and crevicular fluid profile

Determination of the levels of the inflammatory markers TNF-α, IL1-β, IL-6 and IL-8 and IL-10 in the salivary and crevicular fluid will be performed via ELISA, using commercial kits (PeproTech Inc., Rocky Hill, NJ, USA) according to the manufacturer's instructions.

**Figure 1:**
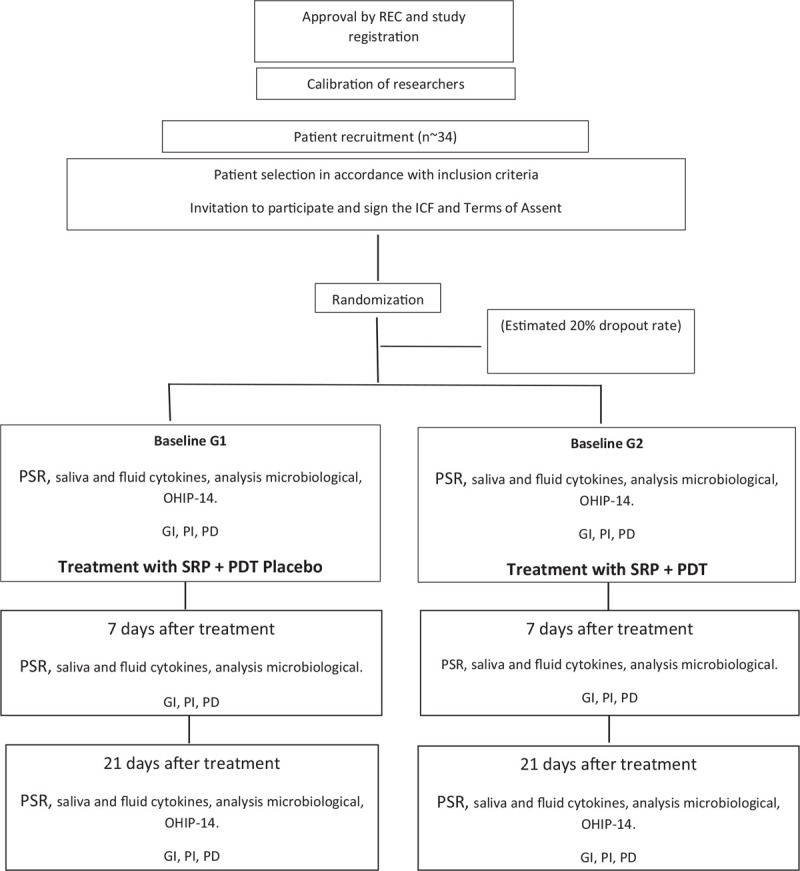
Flowchart. REC = Research Ethics Committee, ICF = Informed Consent Form, PSR = Periodontal Screening and Recording, GI = gingival index, OHIP-14 = Oral Health Impact Profile Questionnaire, PII = plaque index, PD = probing depth, PDT = photodynamic therapy, SSRP = scaling and root planning.

A flowchart is presented for an overview of the study (Fig. 1).

### 2.15 Statistical analysis

Statistical analysis will be performed using only the data obtained from patients who have completed the study. If the sample is normally distributed, Student's T test will be used to compare the means of the continuous and dependent variables. If the distribution is not normal, the Mann-Whitney test will be used. Data will be presented by their means ± SD and the p value will be set at 0.05. All tests will be performed using GraphPad Prism 5 (GraphPad Software, San Diego, USA). The significance level will be set at *p* < 0.05.

Spreadsheets with the survey data will be stored in their own space during the survey. To avoid duplicate data entry, only one researcher will be responsible for entering the data into the spreadsheets for further statistical analysis. The responsible is ACRTH.

## 3 Discussion

Orthodontic treatment has benefits such as esthetics and function, but also some side effects. Considering the necessity of orthodontic treatment for both functional and aesthetic purposes, this long procedure should minimize complications that might make it unfeasible or prolonged. Gingivitis has been identified as one of the possible complications and there have been many studies looking at its prevention during orthodontics. However, it is necessary to discuss therapies to be employed when there is already an imbalance in the buccal system.^[38]^ Establishing therapeutic alternatives for gingivitis during orthodontic treatment, such as a PDT associated with conventional therapy, may improve the patient's quality of life.^[39]^

Fixed orthodontic devices, such as brackets, bands, elastics and their respective dental fixation materials, such as resin and glass ionomer cement, are predisposing factors for biofilm accumulation. Retentive surfaces favor microbiological aggregation and thus development of local inflammatory processes.^[1,2,3,4,5]^ However, this study will not take into consideration bracket type or material, the device used may include accessories and self-bonding; ceramic; steel; or plastic, given that biofilm accumulation can occur on different materials and types.^[6,7]^ The presence of microorganisms in saliva does not seem to present differences related to the specificity of the brackets.^[7,8]^ Jurišić, 2017, states that ceramic brackets are less favorable for biofilm accumulation in adolescents; however, as this study includes children, adolescents and young adults, we did not consider only ceramic brackets.^[9]^

Attention will be given to high quality oral guidance for included patients, recognizing the fact that it is paramount for gum and oral health. The plaque index and bleeding are favored when this measure is taken.^[11,12]^ If the biofilm is not removed regularly, regardless of the treatment or therapies adopted, the dysbiosis will perpetuate, leading to tissue destruction and evolving into a more severe periodontal disease.^[15]^

For the treatment of gingivitis, the gold standard is usually scaling with a curette or ultrasound to remove biofilm.^[20]^ In this study, which considers the challenge of accessing difficult areas, such as in gingival hyperplasia, where the gum may cover the cervical base of the bracket and bands, photodynamic therapy was proposed as an adjuvant treatment, being appropriate for those areas where it is challenging for the curette to reach. The use of ultrasound was disregarded in this study, due to the possibility of bracket detachment with prolonged use, of ultrasound as shown by the Bonetti, 2014.^[10]^

The use of a photosensitizer associated with light at the appropriate wavelength was selected as the adjuvant therapy in this study because it is commonly understood that there is a need to decontaminate sites with gingivitis. PDT has been successfully used to treat periodontal diseases.^[22]^ The PDT ‘therapeutic window’ is effective in reducing the number of colonies or biofilm and may intensify the results of conventional mechanical treatments.^[25,31]^ In addition, while PDT is associated with coronary root scaling in this study, it has been shown to be effective as a primary therapy of choice in hard-to-reach areas, not just as adjunctive therapy.^[1]^

In Alvarenga's study, 2018^[35]^, a 50% attenuation of light at 3 mm from the laser probe was observed. The decrease in light, especially at deep sites where bacteria still remain viable, and the wide range of published results using fiber optics, convinced us to choose the trans-gingival method of application. In addition, a large amount of energy is lost as the fiber apparatus seal is still lacking.

Methylene blue is the most widely used PS in Brazil.^[27,28]^ It was chosen because it is already approved by ANVISA (the Brazilian Health Regulatory Agency), it is easily accessible in the market and it favors clinical applications, as the product is already designed for use in syringes.

Patients will be included according to Chapple, 2018,^[13]^ which characterizes gingivitis as when GI is greater than 10% and probing depth ≤ 3 mm from the distance of the Cemento-enamel Junction to the bottom of the pocket. Clinical signs of gingivitis inflammation will also be considered. Pseudo-pockets will be included in the study, and they will be preferred as choices as they are often areas difficult to access, where PDT can act very effectively.

The randomization of the participants according to the two proposed groups will be followed by the double blinding of the study. The researcher responsible for data collection, the microbiologist and the statistician will be blind to the treatments; the patient will also be blind to the type of treatment performed. We will use the double-blind study methodology to prevent any intentional or unintentional interference with the results of the experiment.

All participants will receive oral hygiene treatment and guidance on the first day of the study. The choice of a single treatment session aims to verify its feasibility in clinical practice, given that orthodontic maintenance is usually performed monthly. Reevaluation after 7 and 21 days will be important to determine if the reduction in inflammation was maintained.

The choice to treat the most contaminated sites rather than the mouth is based on consideration of the oral cavity homeostasis, in which the focus is not on having a totally decontaminated cavity but to have the punctual and local resolution of the inflammation in places difficult to access. To maintain said equilibrium, it is preferably the pseudo-pockets which will be treated with PDT.

The methodology will be determined by a diagnostic and analytical triad: clinical, microbiological and biomarker analysis, considering clinical practice as the most important. We will also evaluate the patient's quality of life as gingival inflammation may cause painful symptoms, loss of function and aesthetic alterations, all or any of which may decrease the patient's quality of life.

In the study by Zanatta et al, 2012,^[39]^ the OHIP14 questionnaire was used to check the quality of life of patients who had gingivitis during fixed orthodontic treatment. It was noticed that there was a greater impact when the patients had anterior gingival enlargement. Thus it was concluded that by preventing and treating gingivitis and anterior gingival enlargement while still undergoing orthodontic treatment, the patient's quality of life could be improved. The OHIP 14 is also the questionnaire of choice for our quality of life assessment.

The gingival index (GI) will be applied according to the dichotomous score,^[34]^ which is presented as a percentage, but it will be considered gingivitis if more than 10% of the sites present bleeding, in accordance with Chapple, 2018.^[13]^ The GI was defined for this study as a primary variable, since its introduction has been widely used in periodontal clinical research and represents the most used index for gingival inflammation in clinical trials with preventive and therapeutic strategies,^[14]^ providing a basis for comparisons between studies.

As biofilm is basically composed of bacteria and your quantity determines the presence of gingivitis, we seek to characterize the disease and not if there is an extra species of microorganism, following Trombelli, 2018,^[14]^ who states that a prominent species is not a pathognomonic sign of gingivitis. Therefore, microbiological analysis of the crevicular fluid and saliva will be performed via universal gene 16SrRNA PCRq at baseline (T0), 7 (T1) and 21 (T2) days after treatment.

Currently biomarkers have also been used in the diagnosis of gingivitis and inflammatory changes whose etiology is due to the presence of non-removable orthodontic devices.^[3]^ Inflammatory cytokines from crevicular fluid and saliva have been evaluated, the evaluation of IL-6, IL-1β, IL-8, TNF-α and IL-10 cytokines in the unstimulated saliva and crevicular fluid by ELISA will be performed at baseline (T0), 7 (T1) and 21 (T2) days after treatment; we have included cytokines with an inflammatory and regulatory profile (IL-10). For Teymouri F, 2016,^[40]^ periodontal treatment with PDT seems to significantly reduce the levels of inflammatory mediators, impacting clinical findings, especially the GI.

Considering that fixed orthodontic treatment is a predisposing factor for biofilm accumulation and consequently for gingivitis,^[38,41]^ it is necessary to introduce innovative therapies without bacterial selectivity, of which PDT is an example,^[25]^ in order to provide uninterrupted orthodontic treatment and maintain the patient's quality of life.^[39]^

## Acknowledgments

We thank thecnical supot of Laboratory of Biophotonics Applied to Health Sciences. of Universidade Nove de Julho, São Paulo, Brazil.

## Author contributions

**Conceptualization**: Anna Carolina Ratto Tempestini Horliana, Ellen Perim Rosa, Sandra Kalil, Lara Motta

**Data curation**: Anna Carolina Ratto Tempestini Horliana, Ellen Perim Rosa, Felipe Murakami Malaquias da Silva, Tania Schalch, Daniela Bezerra Teixeira.

**Formal analysis**: Anna Carolina Ratto Tempestini Horliana, Ellen Perim Rosa, Renata Matalon Negreiros, Daniela Bezerra Teixeira.

**Investigation**: Anna Carolina Ratto Tempestini Horliana, Ellen Perim Rosa, Renata Matalon Negreiros, Priscila Larcher Longo.

**Methodology**: Anna Carolina Ratto Tempestini Horliana, Ellen Perim Rosa, Andre Tortamano, Priscila Larcher Longo.

**Project administration**: Renata Matalon Negreiros, Anna Carolina Ratto Tempestini Horliana, Ellen Perim Rosa, Isabel Peixoto Tortamano.

**Resources**: Anna Carolina Ratto Tempestini Horliana, Ellen Perim Rosa, Ricardo Horliana, Felipe Murakami Malaquias da Silva.

**Supervision**: Renata Matalon Negreiros, Anna Carolina Ratto Tempestini Horliana, Inês Aparecida Buscariolo, Andre Tortamano, Ricardo Horliana.

**Validation**: Anna Carolina Ratto Tempestini Horliana, Sandra Kalil, Lara Motta, Inês Aparecida Buscariolo, Isabel Peixoto Tortamano.

**Writing – original draft**: Anna Carolina Ratto Tempestini Horliana, Ellen Perim Rosa, Renata Matalon Negreiros

**Writing – review & editing**: Ellen Perim Rosa, Renata Matalon Negreiros, Anna Carolina Ratto Tempestini Horliana, Priscila Larcher Longo.

Anna Carolina Ratto Tempestini Horliana orcid: 0000-0003-3476-9064.
